# The effect of diode laser and topical steroid on serum level of TNF-alpha in oral lichen planus patients

**DOI:** 10.4317/jced.52665

**Published:** 2016-12-01

**Authors:** Nagwa-Abdelhamid Othman, Olfat-Gamil Shaker, Hanaa-Mohamed Elshenawy, Wessam Abd-Elmoniem, Amany-Mohy Eldin, Mariam-Yehia Fakhr

**Affiliations:** 1 Prof, Dr. Department of Oral Medicine and Periodontology, Faculty of Oral and Dental Medicine, Cairo University; 2 Prof, Dr. Department of Biochemistry, Faculty of Medicine, Cairo University; 3 Associate, Prof, Dr. Department of Surgery and Oral Medicine, National Research Center; 4 Associate, Prof,Dr. Department of Oral Medicine and Periodontology, Faculty of Oral and Dental Medicine, Cairo University; 5 PhD. Department of Surgery and Oral Medicine, National Research Center; 6 PhD. Department of Oral Medicine and Periodontology, Faculty of Oral and Dental Medicine, Cairo University

## Abstract

**Background:**

Oral lichen planus (OLP) is a common chronic inflammatory mucosal disease with a multifactorial etiology. It is a T-cell mediated autoimmune disease in which the cytotoxic CD8+T cells trigger apoptosis of the basal cells of oral epithelium. Various treatment regimens have been employed for management of symptomatic OLP. This study was carried out to evaluate the effect of topical steroids as well as laser on the clinical signs and symptoms detected by reticular, atrophic, erosive score (RAE score) and tumor necrosis factor- α (TNF-α) level in the serum of patients with symptomatic OLP.

**Material and Methods:**

The study was conducted on twenty-four patients (18 females and 6 males) with symptomatic OLP that were allocated into two groups. Each included twelve patients. The first group treated either with diode laser (970nm SIROLaser Advance class IIIb, SIRONA The Dental Company, Germany) twice weekly with maximum of ten sessions while the second group were treated with topical corticosteroids (0.1% triamcinolone acetonide orabase, Kenacort-A Orabase Pomad, DEVA HOLDING A.Ș, Istanbul, Turkey) for four weeks.

**Results:**

Corticosteroids group showed less clinical signs and symptoms of reticular, atrophic, erosive RAE score (*p*=0.02) and TNF-α serum level (*p*=0.028) than diode laser group with no reported therapy side effects or complications in any of the treated patients.

**Conclusions:**

Topical steroids reduce pain, reticular, atrophic, erosive RAE score and TNF-α serum level more than laser treatment. Moreover, laser treatment can be used as an alternative treatment when steroids are contraindicated for the treatment of symptomatic OLP.

** Key words:**Oral lichen planus, diode laser, topical steroid, RAE score, TNF-α.

## Introduction

Oral lichen planus (OLP) is an inflammatory mucocutaneous disorder of an autoimmune disease. A reported prevalence of 1.27% commonly in women aged between 30 and 60 years (1). In a recent study in Egypt revealed a prevalence of 1.43% among the Egyptians (2). OLP are mostly polymorphic such as reticular type which is present as white striae known as Wickham’s striae often bilateral and asymptomatic. Erosive/atrophic (OLP) present as irregular ulceration, often associated with pain and burning sensation which is exacerbated by trauma, hot, spicy and acidic food (3). OLP pathogenesis is thought to be a T-cell mediated autoimmune disease involving specific and non-specific antigen specificity (4). Antigen specificity includes antigen presentation by basal keratinocytes and antigen-specific keratinocytes by CD8+cytotoxic T-lymphocytes whereas non-specific antigen includes mast cell degranulation and matrix metalloproteinase activation (5). Interleukin-6 (IL-6), Tumor necrosis factor-α (TNF-α) and granulocyte-macrophage colony-stimulating factor (GM-CSF) release and provoke the local inflammatory response (6). Compared with other cytokines TNF-α is the most extensive in OLP, it was consistently observed in OLP when compared with non-lesional or normal oral mucosa (7). Variable treatments were used such as cyclosporine, extracorporeal photochemotherapy, pimecrolimus and tacrolimus (8). Corticosteroids are the main effective drugs of choice for the treatment of symptomatic OLP (9). Topical corticosteroids used in long-time induce insensitivity drug tolerance (10), pseudomembranous candidiasis, and adrenal insufficiency (11). Furthermore, some patients are allergic to corticosteroids while others are insensitive, or even resistant to corticosteroids (12). Recently, novel effective treatments were introduced for treating erosive OLP with minimal side effects as Laser Therapy (13). This study aimed to compare the effect of topical corticosteroid versus diode Laser irradiation in the treatment of oral lichen planus by investigating the serum level of TNF-α.

## Material and Methods

The present study carried out on twenty four patients (18 females, 6 males) with age range from 35-70 years. Patients were recruited from outpatient clinic of Oral Medicine and Periodontology Department, Faculty of Oral and Dental Medicine, Cairo University. The laser group patients were treated in outpatient Dental Clinic in National Research Center. The study protocol was approved by Ethical Committee of Faculty of Oral and Dental Medicine, Cairo University.

-Inclusion criteria

Patients diagnosed with OLP were included in the study based on WHO criteria, if any of them suffered from diabetes and/or hypertension, the condition was controlled before being included in the study.

-Exclusion criteria

Smokers, pregnant and lactating ladies, those with history of topical steroids during the last two months, systemic steroids during the last six months. Patients with uncontrolled diabetes or hypertension, positive HCV Ab or HBs Ag.

-Clinical evaluation

The clinical data were scored according to the criteria scale used by Thongprasom *et al.* (14); score 5: white striae with erosive area >1 cm2, score 4: white striae with erosive area <1 cm2, score 3: white striae with atrophic area >1 cm2, score 2: white striae with atrophic area <1 cm2, score 1: mild white striae only; score 0: no lesions or normal mucosa. The size of the lesions was mea-sured using William’s graduated periodontal probe. For patients with more than one lesion, a sign score was derived by summation of the scores of all areas: Reticular score =Σ R, Atrophic score =Σ A, Erosive/ulcerative score =Σ E with a total weighted score of Σ (R x1)‏ +Σ (A x 1.5) ‏+Σ (E x 2.0) and this score was called RAE score (15). Total improvement (total resolution of the clinical signs) was defined as the disappearance of all atrophic-erosive lesions, regardless of any persisting hyperkeratotic lesions; Partial response, meant a decrease in the patient RAE score compared to baseline; and no improvement defined as no change at all in the patient’s score (16). Patients were categorized into two groups: Group (I): It included twelve patients, eight females and four males with age range of 35-70 years. They were subjected to laser sessions twice weekly. Group (II): It included twelve patients, ten females and two males with age range of 45-62 years. They were treated with topical corticosteroids (0.1% triamcinolone acetonide orabase, Kenacort-A Orabase Pomad, DEVA HOLDING A.Ș, Istanbul, Turkey). Before treatment improvement of the oral hygiene, professional scaling were done for all patients, and oral hygiene instructions were given to reduce the local bacterial load and plaque accumulation. Before laser therapy, normal protective measures were taken. All personnel as well as the patient wore laser safety eye glasses. A gallium-aluminum-arsenide (GaAlAs) diode laser (970nm SIROLaser Advance class IIIb, SIRONA. The Dental Company, Germany) was used. The areas of the lesions were irradiated plus a 3mm margin beyond the visible lesions, using a laser device with 2W irradiation power in a continuous-wave (CW) in a non-contact mode. The exposure time used was eight minutes in four subsequent sessions each with one minute in between to allow for tissue relaxation. The laser beam was delivered using a fiber-optic tip with 320µm diameter. Irradiation was done two times a week for a maximum of ten sessions. After each laser session, a cold diet was recommended and the patient was advised to use chlorhexidine oral gel post-operatively. No side-effects were observed at any time during the treatment and follow-up. In the second group patients were instructed to apply the medication for four times per day, with no food or fluid after administration for a minimum of one hour post-application. They used the medication for maximum of four weeks. Those who extend the treatment to the fourth week were instructed to apply miconazole oral gel to avoid superimposed fungal infection.

-Immunohistochemical analysis

Five milliliter venous blood was withdrawn from both patients’ groups before and after treatment. All blood samples were centrifuged after 30 minutes from their collection. They were then stored in -20°C temperature until laboratory analysis carried on. Quantitation of TNF-α in serum was done by using TNF-α ELISA Kit provided by AviBion, Helsinki FINLAND.

-Statistical analysis

Numerical data were explored for normality by checking the distribution of data, calculating the mean and median values as well as using tests of normality (Kolmogorov-Smirnov and Shapiro-Wilk tests). Age data showed parametric distribution while pain scores and TNF-α levels data showed non-parametric distribution. Data were presented as mean and standard deviation (SD) values. For parametric data; Student’s t-test was used to compare between the two groups. For non-parametric data; Mann-Whitney U test was used to compare between the two groups. Wilcoxon signed-rank test was used to compare between pre- and post-treatment values in each group. Friedman’s test was used to compare between pre-, post-treatment and after exacerbation values in each group. Wilcoxon signed-rank test was used for pair-wise comparisons when Friedman’s test is significant. Bonferroni’s correction was applied for the pair-wise comparisons. Qualitative data were presented as frequencies (n) and percentages (%). Chi-square test was used to compare between the two groups. The significance level was set at *P* ≤ 0.05. Statistical analysis was performed with IBM (IBM Corporation, NY, USA), SPSS (SPSS, Inc., an IBM Company) Statistics Version 20 for Windows.

## Results

-Comparison between the two groups (pre, post treatment and at follow up) regarding the Reticular, Atrophic, Erosive clinical scores.

**Figure 1:**
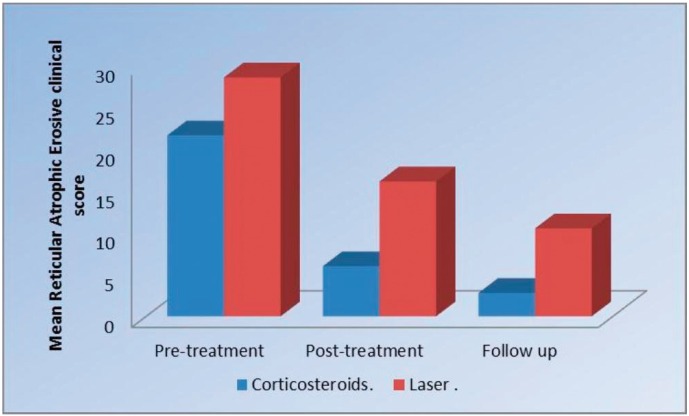
Pre-treatment, there was no statistically significant difference regarding the mean RAE score between the two groups. Post-treatment, Corticosteroids group showed statistically significantly lower mean RAE score than Laser group (*P*= 0.02)

Pre-treatment, there was no statistically significant difference regarding the mean RAE score between the two groups. Post-treatment, Corticosteroids group showed statistically significantly lower mean RAE score than Laser group (*P*= 0.02) (Fig. 1). At follow up, there was no statistically significant difference regarding the mean RAE score between the two groups ( Table 1).

**Table 1:**
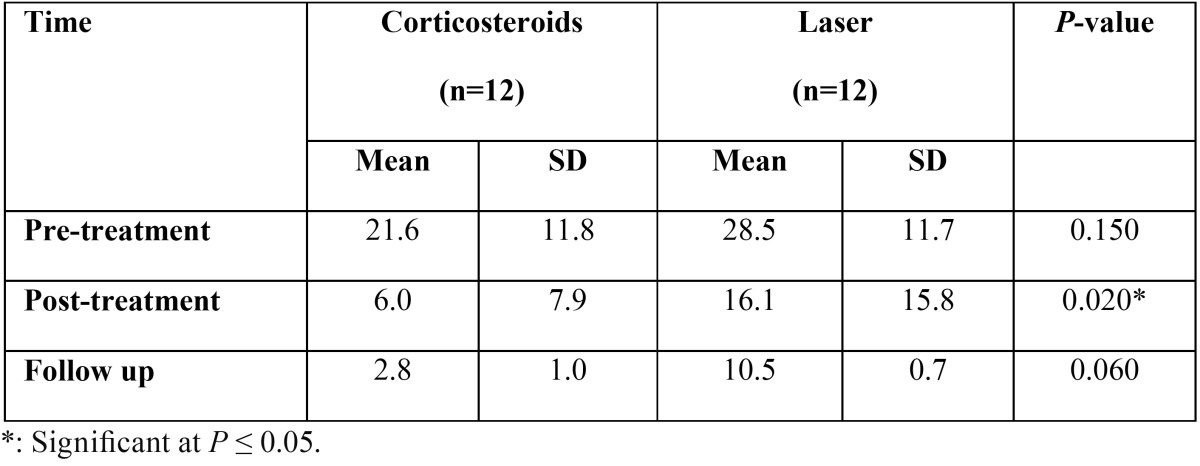
Mean standard deviation (SD) values and results of comparison between RAE clinical scores in the two groups.

-Comparison between the two groups (after exacerbation).

**Figure 2:**
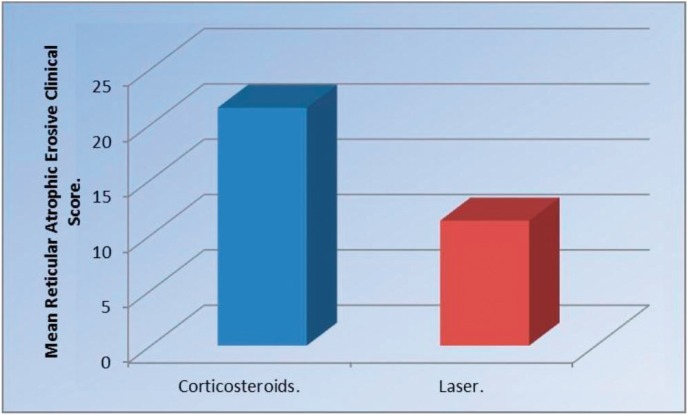
After exacerbation, there was no statistically significant difference between the two groups.

After exacerbation, there was no statistically significant difference between the two groups ( Table 2, Fig. 2).

**Table 2:**
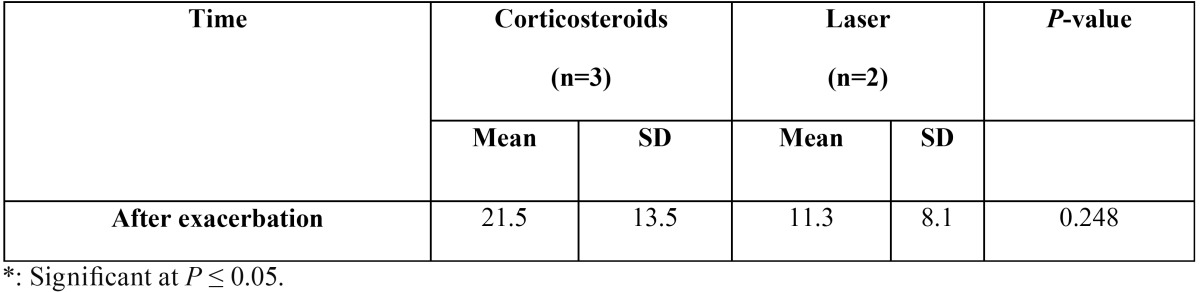
Mean standard deviation (SD) values and results of comparison between Reticular, Atrophic, and Erosive clinical score in the two groups after exacerbation.

-TNF-α levels Comparison between the two groups pre, post treatment and at the follow up.

**Figure 3:**
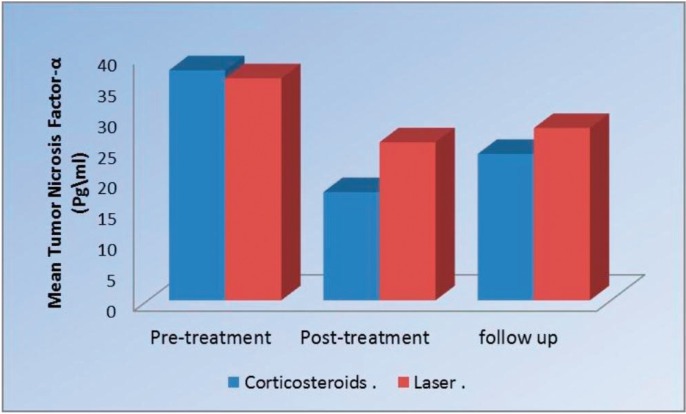
Pre-treatment, there was no statistically significant difference between the two groups. Post-treatment, Corticosteroids group showed statistically significantly lower mean TNF-α levels than Laser group (*P*= 0.028).

Pre-treatment, there was no statistically significant difference between the two groups. Post-treatment, Corticosteroids group showed statistically significantly lower mean TNF-α levels than Laser group (*P*= 0.028) (Fig. 3). At follow up, there was no statistically significant difference between the two groups ( Table 3).

**Table 3:**
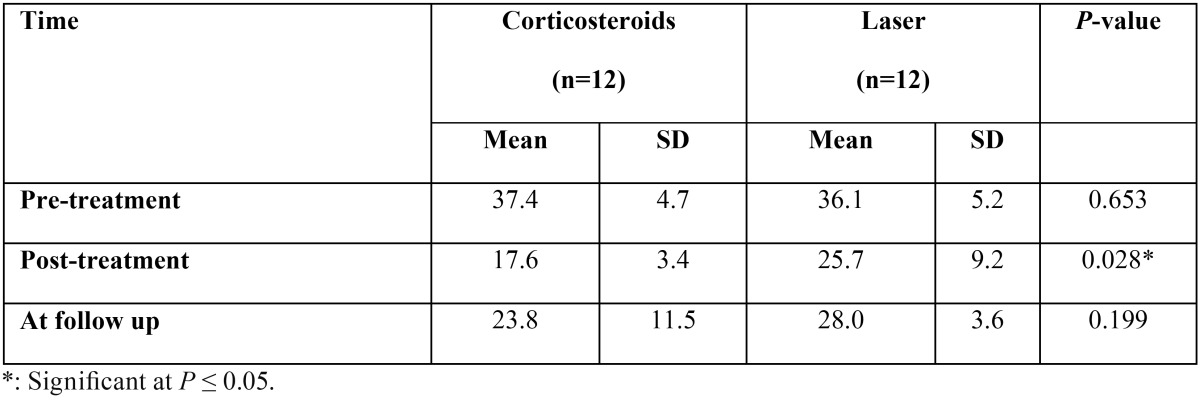
Mean standard deviation (SD) values and results of comparison between TNF-α level in the two groups.

## Discussion

Etiologic factors causing OLP are genetic factors, autoimmune, infectious agent, stress, diabetes, hypertension and malignant neoplasm (5). Corticosteroids are the most common group of drugs for the treatment of symptomatic OLP due to the fact of their ability to modulate inflammation and immune response by reducing the lymphocytic exudate and stabilization the lysosomal membrane (17). OLP patients unresponsive to topical superpotent corticosteroids, surgical management using cryosurgery and different types of laser were tried. A 980-nm diode laser; CO2 laser evaporation (18); low-dose excimer 308-nm laser with UV-B rays (19) and biostimulation with a pulsed diode laser using 904-nm pulsed infrared rays have been tried (20). It was believed that laser acts by local stimulation of the diseased tissues by improving the local microvasculature (21), reducing the amount of inflammatory mediators, increasing cellular activity, increasing the quantity of granulation tissue and hence, stimulating the normal physiologic healing process (22). This may be attributed to stimulation of vascular smooth muscles relaxation (22). It is postulated that laser enhances β-endorphin and encephalin release in tissues, inhibits prostaglandin E2 production, and improves blood flow and lymphatic drainage, and decrease edema (23), many recent studies are investigating the application of LLLT for treatment of OLP (24). The wavelength selected was 970-nm since it allows superficial action from an optical point of view. The exposure time used was 4 minutes; this duration was decided after pilot study on four patients. It aimed to achieve maximum benefit without any laser side effects. The laser group of patients assisted 2 sessions weekly as recommended by Kazancioglu *et al.* (24). The serum TNF-α level is proposed to increase in OLP patients in contrast to healthy people (25); and therefore TNF-α was evaluated in this study and considered as an indicator for OLP disease activity. Several studies have proved the analgesic effect of soft laser in acute (25) and chronic pain of various etiopathogenesis (26). It is well known that corticosteroids reduce pain by the inhibitory effect on the phospholipase enzyme, preventing the conversion of phospholipids into arachidonic acid critical for prostaglandins production (27). In the laser group, statistically significant decrease in RAE post treatment (*p*=0.035) was found. This is coinciding with the results of Cafaro *et al.* (20) who showed significant clinical improvement (*p*=0.001). In this study, the group treated with topical steroids showed also statistically significant reduction in the RAE scores (*p*= 0.018). Nevertheless, topical steroids group showed statistically significant lower mean scores than the laser group (*p*=0.02). In this study, low power laser improved microcirculation and promoted angiogenesis (25); this may explain the clinical improvement achieved in patients treated with laser. On the other hand, improvement in the steroids group mostly depends on the inhibition of the inflammatory process of the disease allowing for physiologic tissue repair (27).
